# Anticoagulation Stewardship in the Era of Practice-Changing Evidence: A Narrative Review of Recent Trials and Their Bedside Translation

**DOI:** 10.3390/jcm15155946

**Published:** 2026-07-30

**Authors:** Mansour Aljabry, Ghazi Alotaibi

**Affiliations:** 1 Department of Pathology and Laboratory Medicine, College of Medicine, King Saud University, Riyadh 11451, Saudi Arabia; 2 Department of Medicine, College of Medicine, King Saud University, Riyadh 11451, Saudi Arabia; galo@ksu.edu.sa

**Keywords:** anticoagulation stewardship, direct oral anticoagulants, factor XI inhibitors, translation gap, medication safety, narrative review

## Abstract

**Background/Objectives:** The evidence base for anticoagulation moved substantially between 2023 and 2026: a reframed atrial fibrillation (AF) guideline, new randomized data on extended treatment of cancer-associated venous thromboembolism (VTE), the first adequately powered head-to-head comparison of two direct oral anticoagulants (DOACs), the first randomized evidence for andexanet alfa, and mixed results for the factor XI inhibitor class. Real-world uptake remains incomplete. To review the 2023–2026 evidence changing anticoagulation practice, identify where bedside practice lags behind it, and outline the institutional adjustments needed to translate it into routine care. **Methods:** Narrative review. PubMed, EMBASE, and the Cochrane Library were searched from 2000 to January 2026, with hand-searching of society guidelines and regulatory documents; eligible sources were randomized trials, pre-specified subgroup analyses, prospective registries, systematic reviews, and society-endorsed guidelines. A targeted update in March 2026 captured practice-changing trials published after the primary search date, identifying COBRRA (published 11 March 2026). This is a narrative, non-systematic review reported in accordance with SANRA: no protocol was registered, topic selection is interpretive, and no formal risk-of-bias assessment or quantitative synthesis was undertaken. Recommendations are labeled by evidence basis. **Results:** Seven developments warrant attention: (1) the 2023 AF guideline’s ≥2%/year anticoagulation threshold, with sex-specific application and a 1–2%/year shared decision zone; (2) API-CAT—reduced-dose apixaban non-inferior to full dose for extended cancer VTE prevention (2.1% vs. 2.8%; adjusted subhazard ratio 0.76, 95% CI 0.41–1.41; pre-specified non-inferiority margin 2.00), with less bleeding (12.1% vs. 15.6%); (3) COBRRA—apixaban substantially safer than rivaroxaban in acute non-cancer VTE (clinically relevant bleeding 3.3% vs. 7.1%; RR 0.46, 95% CI 0.33–0.65; major bleeding 0.4% vs. 2.4%; RR 0.16, 95% CI 0.06–0.40), without efficacy trade-off; (4) ANNEXA-I—a modest hemostatic advantage for andexanet alfa over usual care (67.0% vs. 53.1%) at the cost of increased thrombosis (10.3% vs. 5.6%); (5) an asymmetric factor XI landscape in which class-level conclusions remain premature; (6) new special population data (ELDERCARE-AF, AZALEA sub-analyses by kidney function); and (7) a maturing but uneven stewardship evidence base. **Conclusions:** Three years of trials have produced concrete implications for how anticoagulation should be practiced now. The interval between publication and routine implementation remains the largest practical problem in the field, and institutional stewardship structures are a major mechanism for closing it.

## 1. Introduction: The State of Anticoagulation Practice

Anticoagulants are among the most frequently prescribed drug classes in hospital practice and are simultaneously among the most frequent causes of drug-related harm. Both effects are large, and they are produced by the same prescriptions. In United States pharmacovigilance data, anticoagulants are the drug class most frequently implicated in emergency department visits for adverse drug events, accounting for 17.6% of such visits across all ages, for 27.5% among adults aged 65 to 79, and for 38.8% among those aged 80 and older; analyses of the 2017–2019 period confirm a persistent pattern [1,2]. These are United States surveillance data; equivalent national pharmacovigilance reporting does not exist for most other health systems, and the proportions should not be assumed to transfer directly to settings with different prescribing patterns, population age structures, and case ascertainment methods. Much of the harm that accrues in any given week of hospital practice emerges not from obvious errors but from small, cumulative mismatches between prescribing decisions and the changing physiological state of the patient—apixaban doses reduced on the basis of a single label criterion, missed drug interactions, unreconciled renal function changes. These represent system-related prescribing failures and failures in medication management within the institutions where prescriptions are issued, rather than shortcomings of the drugs themselves.

The direct oral anticoagulants (DOACs)—dabigatran, rivaroxaban, apixaban, edoxaban—were expected to reduce this burden with fixed dosing, predictable pharmacokinetics, and no routine monitoring [3,4,5,6]. A decade of real-world evidence has delivered a more complicated verdict. Intracranial hemorrhage rates have fallen, but dose-inappropriate prescribing has risen, and a new spectrum of harm has emerged from unrecognized drug interactions, missed renal adjustments, and fragmented transitions of care [7,8,9]. Prescribing the DOACs became simpler; the workflows surrounding them did not.

Anticoagulation stewardship—coordinated, multidisciplinary oversight of anticoagulant use—is the institutional response to this mismatch. Professional societies have championed it for more than a decade [10,11,12,13,14,15]. This review examines where the science of anticoagulation has moved between 2023 and 2026, where stewardship programs operate most consequentially, and what modest policy adjustments would accelerate translation of scientific advances into routine care. Clinical medicine of this complexity is not delivered by individual expertise alone. It is delivered through organizational systems—stewardship structures, decision support, and the accreditation and payment frameworks that shape them—and the trials reviewed here reach patients only to the extent that those systems carry them. We therefore treat the clinical science and the institutional machinery that delivers it as inseparable rather than as a major and a minor partner in the argument. The scope and structure follow one division. Section 3, Section 4 and Section 5 synthesize the evidence: the burden of anticoagulant-related harm, the functions of stewardship, and the practice-changing trials and guidelines of 2023 to early 2026. Section 6, Section 7, Section 8 and Section 9 are different in kind, setting out institutional and policy proposals that follow from that evidence but are not established by it, labeled accordingly under the grading scheme in Section 2.4; a reader wanting the evidence synthesis alone may read Section 3, Section 4 and Section 5 and stop. We chose the 2023 to 2026 window because it opens with the 2023 ACC/AHA/ACCP/HRS atrial fibrillation guideline, which reset the anticoagulation decision itself, and because the interval since has delivered an unusually dense run of practice-changing trials—API-CAT, COBRRA, ANNEXA-I, OCEANIC-AF, AZALEA-TIMI 71, and OCEANIC-STROKE—not previously reviewed together for their combined effect on bedside practice. Earlier evidence is cited where it remains the basis of current practice but is not re-reviewed.

## 2. Methods

This article is a narrative review. It is not a systematic review, and it should not be read as one. No protocol was registered (for example, with PROSPERO), no reproducible search string was applied across all included topics, study selection was not duplicated by independent reviewers, and no formal risk-of-bias assessment or quantitative evidence synthesis was undertaken. Four measures were adopted to limit, though not to eliminate, the risk of selection bias that follows from this design. First, the criterion for including a development was fixed in advance and applied uniformly, so that topics were not admitted merely because they were novel or heavily cited. Second, every effect estimate was taken from the primary publication rather than a secondary summary, so that the evidence was not filtered through another author’s emphasis (Section 2.3). Third, evidence that runs against our argument is reported explicitly rather than omitted—the OCEANIC-AF failure, the thrombotic signal in ANNEXA-I, the numerically higher ischemic stroke rate with abelacimab in AZALEA-TIMI 71, and the inconclusiveness of the dialysis trials are each stated in the sections concerned. Fourth, every recommendation carries a label identifying the kind of evidence it rests on (Section 2.4), so that a reader can discount our interpretive claims independently of our evidential ones. The purpose of the review is interpretive: to identify developments between 2023 and 2026 with direct implications for bedside anticoagulation practice, and to describe the institutional structures through which those developments reach patients. We set out our sources and selection process below so that readers can judge the basis of each statement, while recognizing that a narrative synthesis of this kind carries a risk of selection bias that a systematic review is designed to minimize. The article is structured in accordance with SANRA (the Scale for the Assessment of Narrative Review Articles), the reporting instrument developed for non-systematic reviews, rather than PRISMA [16]. The account of sources below should therefore be read as a transparent description of how evidence was identified, weighed, and verified—SANRA items 3 and 4—and not as a reproducible search protocol. We have deliberately avoided presenting it in the apparatus of a systematic review, because doing so would imply a completeness of retrieval that we did not attempt and cannot claim.

### 2.1. Sources and Search

We searched PubMed, EMBASE, and the Cochrane Library from 2000 to January 2026, using combinations of the terms “direct oral anticoagulant,” “factor XI inhibitor,” “andexanet,” “anticoagulation stewardship,” “atrial fibrillation,” and “cancer-associated thrombosis.” Society guidelines, regulatory documents, and accreditation standards were hand-searched, and reference lists of retrieved articles were screened for additional sources. A targeted update was subsequently performed in March 2026 to capture practice-changing trials published after the primary search date; this update identified the COBRRA trial, published on 11 March 2026, which is discussed in Section 5.3. The update was targeted at practice-changing randomized trials in the domains already covered rather than being a repeat of the full search.

### 2.2. Eligibility and Selection

Eligible sources were randomized controlled trials, pre-specified subgroup and secondary analyses of randomized trials, prospective registries, systematic reviews and meta-analyses, and society-endorsed guidelines. Narrative commentary, conference abstracts without subsequent full publication, and press releases were not used as the basis for clinical recommendations; where a trial result was available only as a sponsor announcement at the time of writing, this is stated explicitly in the text and no recommendation is drawn from it. Selection of the developments highlighted was made by consensus between the two authors against a single criterion: whether the finding should change what a clinician or a stewardship program does, rather than novelty or citation profile. Both authors reviewed the candidate developments independently against that criterion and then compared their lists; disagreements were resolved by discussion until consensus was reached, and no third adjudicator was used. We record this as a limitation rather than as a procedure: two authors reaching agreement is not the same safeguard as duplicate screening against a pre-specified protocol, and it does not protect against a bias the two of us share. Topics with an established evidence base that has not materially changed since 2023 were therefore excluded, as were developments confined to pre-clinical or early-phase work. This selection is interpretive, and other authors applying the same criterion might reasonably have chosen differently.

### 2.3. Data Verification

For every trial discussed, effect estimates, confidence intervals, denominators, and dosing regimens were taken from the primary publication rather than from secondary summaries, and were checked against the published tables. Bibliographic details were verified against the journal record, and trial registration numbers are given in the reference list where available. Where our reading of a trial differs from a common secondary characterization of it, we state the primary figure.

### 2.4. Evidence Grading

Because a narrative review can otherwise present claims of very different provenance in the same register, recommendations in this article are labeled according to their basis: [RCT] for statements following directly from randomized evidence; [Guideline] for statements reproducing a society-endorsed recommendation; [Registry] for statements resting on non-randomized prospective data; [Institutional interpretation] for operational inferences we draw from evidence but which no trial or guideline states; and [Expert opinion] for the authors’ judgment. Labels appear against the recommendations in Section 3, Section 4 and Section 5, Section 7 and Section 9 and in the Key Points box. Readers should weigh the last two categories accordingly; they represent our view of how the evidence should be operationalized, not evidence itself.

## 3. The Clinical Burden of Anticoagulant-Related Harm

The visible burden of anticoagulant harm is substantial. Major bleeding rates in contemporary DOAC AF cohorts run at 2–4% per patient-year, with intracranial hemorrhage at 0.3–0.5% per year—roughly half the warfarin rate but still clinically significant [3,4,5,6,17], and two- to three-fold higher in patients over 80 and those with renal impairment [18]. Pooled registry estimates mask substantial heterogeneity across populations and agents; generalizability to Middle Eastern or South Asian populations remains poorly established. These ranges should be read as conditional rather than universal. They derive predominantly from North American and European trial and registry cohorts, and bleeding and intracranial hemorrhage risk vary with ethnicity, background stroke and bleeding risk, comorbidity burden, and the quality of anticoagulation management; no contemporary surveillance of comparable size exists for most Middle Eastern, African, or South Asian populations, which is itself a finding worth stating plainly.

Three categories of harm are systematically undercounted (Table 1). First, errors of omission: registry data suggest that 30–40% of AF patients with guideline-indicated anticoagulation do not receive it at therapeutic doses. This figure is strongly setting-dependent and should not be pooled across contexts: it derives principally from PINNACLE, a United States national outpatient cardiology practice registry, and from comparable specialist cohorts, whereas community and primary care series report consistently worse performance and highly selected tertiary referral practice reports better. The gap is neither confined to North America nor to atrial fibrillation. In the AVAIL ME Extension survey—a cross-sectional study of 4983 patients across 101 hospitals in ten Middle Eastern and Central Asian countries—3575 patients (72%) were judged eligible for venous thromboembolism prophylaxis and 2747 (77%) of those received some drug prophylaxis, yet overall concordance with ACCP guidelines was only 38%, and just 24% among medical patients against 44% among surgical patients [Registry] [19]. Regional data of this kind are scarcer than they should be, and where they exist, they show the same structural failure the stewardship literature describes in high-income settings. Second, inappropriate DOAC dose reduction—the “less-is-safer” intuition—associates consistently with increased thromboembolism and no offsetting bleeding reduction; the direction of the signal is reliable, though residual confounding probably inflates the magnitude reported in observational studies. Third, and most neglected, transition-of-care failures: duplicate therapy, failed post-procedure resumption, and patients lost to follow-up after VTE discharge fall between inpatient and outpatient surveillance. Mandatory reporting of dose-appropriateness rates, transition-of-care outcomes, and equity-stratified prescribing would expose what current metrics obscure; these outcomes are under-measured because reporting has not been required, not because measurement is infeasible.

## 4. What Stewardship Does—And Where It Matters Most

**Figure 1:**
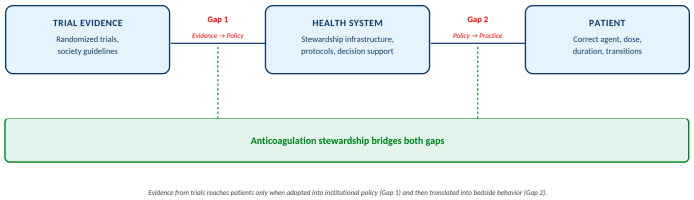
The translation gap in anticoagulation practice. Stewardship programs intervene at both the evidence-to-policy (Gap 1) and policy-to-practice (Gap 2) junctions. HTA, health technology assessment.

Anticoagulation stewardship is coordinated, multidisciplinary, institution-wide oversight of anticoagulant selection, dosing, monitoring, duration, transitions, and reversal. Its structure is modeled on antimicrobial stewardship but adapted to a fundamentally different pharmacology [15,20]. Conceptually, stewardship functions as the connective infrastructure between published trial evidence and bedside practice (Figure 1). Two translation gaps separate trial publication from patient benefit: an upstream gap in which trial results must be absorbed into institutional policy, and a downstream gap in which policy must become consistent bedside behavior. Much of the 2023–2026 evidence reviewed below is currently stalled at one or both junctions.

A mature program requires five organizational elements—physician lead (typically a hematologist with thrombosis expertise), clinical pharmacists with anticoagulation training, nursing leadership, clinical informatics partners, and formal linkage to quality and safety committees—and operates across nine functional domains: drug selection, dose optimization, monitoring, duration review, transitions of care, peri-procedural management (codified by BRIDGE for warfarin and PAUSE for DOACs) [21,22], bleeding and reversal, education, and metrics. Integration across domains is what defines a program; each in isolation is evidence-backed but insufficient. Registry data complement these trials in settings they did not address: the Italian IRIS registry, a prospective multicenter non-interventional study of 250 consecutive patients with atrial fibrillation receiving rivaroxaban and undergoing catheter ablation, reported real-world outcomes with uninterrupted versus briefly interrupted rivaroxaban, and illustrates the peri-procedural practice variation that stewardship programs are designed to standardize; as a non-randomized registry, it informs rather than determines protocol content [Registry] [23].

Not every anticoagulation decision requires stewardship oversight. Marginal benefit is concentrated at a small number of high-stakes decision nodes (Table 2) where errors are common and consequential; prioritizing these allows resource-constrained programs to operate rationally.

One example illustrates the clinical depth and operational complexity at once. A 72-year-old woman is started on apixaban for non-valvular AF. She weighs 58 kg, baseline creatinine 1.3 mg/dL (CrCl~40 mL/min), taking amiodarone after failed rhythm control. The label permits full-dose apixaban (she meets only one of three reduction criteria: weight ≤ 60 kg). Nonetheless, in audits we are familiar with, a substantial minority of clinicians reduce the dose on gestalt concern about bleeding—a pattern associated with increased thromboembolism without reducing bleeding. Amiodarone raises apixaban exposure modestly but does not by itself warrant dose reduction per ISTH guidance. Eighteen months later, her creatinine drifts to 1.6 mg/dL during a dehydrating illness, but nothing in the system flags the dose for review. Six weeks after that, she presents with a gastrointestinal bleed. Each step was individually defensible; the outcome was avoidable with a stewardship infrastructure that (a) flagged the one-of-three criteria at prescribing, (b) documented the amiodarone interaction, and (c) triggered automated review when the creatinine changed. Versions of this case recur at morbidity and mortality rounds across many centers; the question of which structural intervention reduces such events most efficiently rests on comparative evidence still thinner than it should be.

## 5. Where the Science Is Moving: Clinical Updates 2023–2026

This review is structured around the clinical question it seeks to help readers answer: what has changed in how anticoagulation should be practiced between 2023 and early 2026, and how should a well-organized department translate those changes into routine care? We consider seven developments in turn: the 2023 ACC/AHA/ACCP/HRS atrial fibrillation guideline (Section 5.1), the API-CAT trial for extended cancer VTE prevention (5.2), the COBRRA trial comparing apixaban with rivaroxaban in acute VTE (5.3), the maturation of the andexanet alfa evidence base following ANNEXA-I (5.4), the emerging—and ambiguous—factor XI inhibitor class (5.5), updated guidance in special populations (5.6), and the evidence base for stewardship programs themselves (5.7), which has matured alongside the drug science at a slower pace. Table 3 summarizes these developments and the practical implication of each.

### 5.1. The 2023 AF Guideline: From Score-Based to Risk Magnitude Thresholds

The 2023 ACC/AHA/ACCP/HRS atrial fibrillation guideline [24] reshaped the anticoagulation decision in a way that stewardship programs must operationalize. Rather than anchoring the decision exclusively on the CHA_2_DS_2_-VASc score cut-off, the guideline anchors the recommendation on the magnitude of annual thromboembolic risk and stratifies into three zones: ≥2%/year (Class I recommendation for anticoagulation), 1–2%/year (shared decision, Class IIa), and <1%/year (no routine anticoagulation, Class III). Validated risk calculators beyond CHA_2_DS_2_-VASc—including ATRIA and GARFIELD-AF—are endorsed, and clinical risk modifiers (AF burden, persistent vs. paroxysmal pattern, obesity, hypertrophic cardiomyopathy, renal function, proteinuria, left atrial size) are brought into play for patients in the intermediate 1–2%/year zone. The guideline also redefines female sex as a risk-modifying rather than independent risk factor, with implications for the anticoagulation threshold in women (effectively CHA_2_DS_2_-VASc ≥ 3 rather than ≥2).

For a stewardship program, the operational implications are concrete: decision support must present annual risk magnitude rather than a raw score; prescribers need explicit guidance for the 1–2%/year intermediate zone; and sex-specific thresholds require careful implementation to avoid both under- and over-treatment of women. Uptake of the 2023 changes remains uneven even in high-income settings and slower where the guideline translation process is delayed.

### 5.2. API-CAT: Extended Reduced-Dose Apixaban for Cancer VTE

The API-CAT trial (Mahé et al., NEJM 2025) [25] addressed a long-standing uncertainty: what to do with cancer-associated VTE patients beyond the first six months of anticoagulation. The trial randomized 1766 patients with active cancer and prior proximal DVT or pulmonary embolism who had completed at least six months of anticoagulation to continued full-dose apixaban (5 mg twice daily) versus reduced-dose apixaban (2.5 mg twice daily) for a further twelve months. API-CAT was a randomized, double-blind non-inferiority trial with blinded central outcome adjudication. The primary efficacy outcome was centrally adjudicated fatal or non-fatal recurrent venous thromboembolism, with non-inferiority pre-specified as an upper boundary of the 95% confidence interval of the subhazard ratio below 2.00. Reduced-dose apixaban met that criterion (cumulative incidence 2.1% versus 2.8%; adjusted subhazard ratio 0.76, 95% CI 0.41–1.41; *p* = 0.001 for non-inferiority). The confidence interval is wide, reflecting a low absolute event rate, and the result should be read as compatibility with a pre-specified margin rather than as demonstrated equivalence. The key secondary outcome, clinically relevant bleeding, was assessed in a superiority analysis and favored the reduced dose (12.1% versus 15.6%; adjusted subhazard ratio 0.75, 95% CI 0.58–0.97)—a clinically attractive profile for a population in whom the thrombotic risk persists but the bleeding risk, over time, becomes the more constraining consideration.

API-CAT does not obviate shared decision-making—patients with progressing malignancy, central venous catheters, or recent thrombosis may still benefit from full-dose continuation—but supports a defensible default. Stewardship programs should update cancer VTE protocols to prompt dose reassessment at six months and document the basis for continuation versus de-escalation.

### 5.3. COBRRA: Apixaban Versus Rivaroxaban in Acute VTE

For more than a decade after DOAC approval, the choice between apixaban and rivaroxaban for acute VTE rested on indirect comparisons. The COBRRA trial (Castellucci et al., NEJM 2026) [26] provided the first adequately powered randomized head-to-head comparison. A total of 2760 patients with acute symptomatic pulmonary embolism or proximal deep vein thrombosis were randomized to apixaban (10 mg twice daily for 7 days, then 5 mg twice daily) or rivaroxaban (15 mg twice daily for 21 days, then 20 mg once daily) for three months. The primary outcome—clinically relevant bleeding (ISTH major or clinically relevant non-major)—occurred in 3.3% of the apixaban group and 7.1% of the rivaroxaban group (relative risk 0.46, 95% CI 0.33–0.65). Major bleeding was reduced from 2.4% to 0.4% (RR 0.16, 95% CI 0.06–0.40). Recurrent VTE rates did not differ. Cancer-associated VTE was excluded, so the API-CAT logic for that population is unaffected.

Two design points bear on how strongly this result should be read. COBRRA was an investigator-initiated, open-label trial with blinded endpoint adjudication (PROBE design), and its primary bleeding endpoint was tested under a superiority hypothesis rather than for non-inferiority; the finding is therefore a positive demonstration that apixaban is safer, not merely evidence that it is no worse. It is also, more precisely, the first adequately powered randomized head-to-head comparison of two direct oral anticoagulants in acute venous thromboembolism: smaller randomized comparisons and comparisons in narrower or non-registrational settings have been reported before, and the claim we make rests on scale and on the pragmatic, practice-level question the trial was designed to answer rather than on absolute primacy. The accompanying editorial reports that 26 patients would need to be treated with apixaban rather than rivaroxaban to avoid one clinically relevant bleeding event, and 53 to avoid one major bleeding event. The trial investigators note that the difference may reflect rivaroxaban’s higher early-phase exposure (30 mg/day for 21 days), since the outcomes diverged mainly during the first three weeks, and that whether the dosing regimen accounts for the observed bleeding risk requires further evaluation. The bedside implication follows from randomized evidence [RCT], though it rests on a single trial and we frame it accordingly: on current evidence, apixaban may reasonably be preferred over rivaroxaban as the default DOAC for acute non-cancer VTE, unless formulary, renal, adherence, or patient-specific considerations indicate otherwise. We stop short of a categorical recommendation. COBRRA is a well-conducted and adequately powered trial, but it is one trial, it was open-label, it did not enroll patients with cancer-associated thrombosis, and its findings have not yet been incorporated into society guidance; a recommendation of this reach should await either guideline endorsement or corroboration. Updating institutional order sets and emergency department DOAC pathways accordingly is our institutional interpretation of that evidence rather than a guideline recommendation [Institutional interpretation]. COBRRA also reopens the formulary question for health systems that have negotiated volume-based pricing contracts favoring rivaroxaban; the accompanying editorial argues that these results should prompt a re-evaluation of current guidelines [Expert opinion] [26].

### 5.4. Andexanet Alfa After ANNEXA-I: Resolution with Residual Uncertainty

ANNEXA-I (Connolly et al., NEJM 2024) [27] was the first randomized trial of andexanet alfa versus usual care in factor Xa inhibitor-associated acute intracerebral hemorrhage. It was a phase 4, multicenter, prospective, randomized, open-label trial with blinded endpoint adjudication (PROBE design), enrolling 530 patients at 131 sites in 23 countries. The design matters to interpretation: the primary endpoint of hemostatic efficacy is a composite of hematoma expansion, change in NIHSS score, and receipt of rescue therapy, and the latter two components are management-dependent and only partially objective. Central adjudication masked to treatment assignment mitigates that exposure but does not remove it, and a soft composite endpoint in an unblinded trial warrants more caution than an equivalent effect size on a hard endpoint would. In practice, 85.5% of the usual care group received 4F-PCC, making this effectively a comparison of andexanet against 4F-PCC rather than against no reversal. The primary hemostatic efficacy endpoint favored andexanet (67.0% vs. 53.1%), but thrombotic events were more common in the andexanet group (10.3% vs. 5.6%; ischemic stroke 6.5% vs. 1.5%), and the trial was stopped early for efficacy rather than for an overall benefit–harm demonstration. At approximately US$50,000 per full dose—roughly ten times the cost of 4F-PCC—the benefit–cost–risk calculation is unavoidably complex.

A restricted use pathway is a reasonable institutional response [Institutional interpretation]: andexanet for ANNEXA-I-eligible intracerebral hemorrhage within the dosing window, and 4F-PCC for extracranial major bleeding and for patients outside andexanet eligibility. In our view, this rational institutional response also illustrates a wider problem: in the absence of national guidance, decisions of this cost and consequence are in practice being taken hospital by hospital. We advance this as our reading of the situation rather than as an established finding [Expert opinion]; it is consistent with the divergence in national health technology assessment positions described in Section 8, where the United Kingdom, Germany, and Canada have issued restrictive guidance and the United States has not. The andexanet situation contrasts with the dabigatran reversal story, in which idarucizumab achieved hemostatic control without a comparable thrombotic signal [30]—within-class reversal agent properties do not extrapolate across anticoagulant classes.

### 5.5. Factor XI Inhibitors: A Class That Has Surprised on Both Sides

The factor XI/XIa inhibitor class has been the most anticipated new entry in the anticoagulation space for a decade. The scientific rationale is that factor XI contributes more to pathological thrombosis than to physiological hemostasis, offering the theoretical possibility of anticoagulation with lower bleeding risk. Three agents have advanced furthest, and they are not pharmacologically equivalent. Abelacimab is a fully human monoclonal antibody that binds the catalytic domain of factor XI and locks the zymogen in its inactive state, preventing generation of factor XIa while also inhibiting factor XIa itself; it is given by monthly subcutaneous injection and has a correspondingly long half-life. Milvexian and asundexian are orally administered small molecules that inhibit the activity of factor XIa alone. Target (zymogen versus activated enzyme), route, half-life, and achieved potency therefore differ across the three agents, and these differences are material to interpreting the trials summarized below (Table 4).

Results to date have been mixed, but the two headline trials are asymmetric by design and the word “bifurcated” should not be taken to imply that they are commensurate. OCEANIC-AF was a large phase 3 trial powered for efficacy against an active comparator; AZALEA-TIMI 71 was a phase 2b bleeding event-driven trial whose sample size and stopping rule were built around a safety endpoint and which was neither planned nor powered to demonstrate stroke prevention. A safety success in the second is not an efficacy counterweight to a failure in the first, and the two should not be placed on the same evidential footing. The phase 3 OCEANIC-AF trial of asundexian versus apixaban in AF (Piccini et al., NEJM 2025) [28] was stopped early for inferiority of asundexian (hazard ratio for stroke or systemic embolism 3.79). Conversely, the phase 2b AZALEA-TIMI 71 trial of abelacimab versus rivaroxaban (1287 patients randomized; median follow-up 2.1 years) was stopped early by its data monitoring committee for a greater-than-anticipated reduction in bleeding (Ruff et al., NEJM 2025) [31]: at the 150 mg dose, major or clinically relevant non-major bleeding was reduced by 62% (hazard ratio 0.38; 95% CI 0.24–0.60), major bleeding by 67% (hazard ratio 0.33; 95% CI 0.16–0.66), and major gastrointestinal bleeding by 89% (hazard ratio 0.11; 95% CI 0.03–0.48). A pre-specified analysis showed consistent safety across strata of kidney function [32]. AZALEA-TIMI 71 was powered for safety rather than efficacy, and stroke and systemic embolic events were few. Ischemic stroke was numerically more frequent with abelacimab than with rivaroxaban (150 mg dose: hazard ratio 2.06; 95% CI 0.70–6.02), an imprecise estimate that the trial was neither designed nor powered to evaluate. AZALEA-TIMI 71 therefore does not establish that abelacimab matches rivaroxaban for stroke prevention, and this uncertainty—not the bleeding result—is what the phase 3 program must resolve.

Phase 3 results will determine the class’s future (Table 4). LIBREXIA-AF is comparing milvexian 100 mg twice daily with apixaban, a four-fold higher total daily dose than the asundexian regimen that failed in OCEANIC-AF, and milvexian is additionally more potent than asundexian in in vitro factor XIa inhibition assays; it has enrolled more than 20,000 participants and remains ongoing, with topline data anticipated in 2026. LILAC-TIMI 76 is evaluating abelacimab against placebo in approximately 1900 AF patients aged 65 years or older deemed unsuitable for conventional anticoagulation, with completion anticipated in the second half of 2026. OCEANIC-AFINA was announced in November 2023 to evaluate asundexian against placebo in a comparable OAC-ineligible population. Nine days later, when OCEANIC-AF was stopped, the sponsor stated that recruitment for OCEANIC-AFINA had not begun and that the design would be re-evaluated in light of that result [28]. As of our final search in March 2026, we identified no record that recruitment had started, and we therefore make no claim that the trial is active. The wider class program has since bifurcated further. LIBREXIA-ACS was stopped in November 2025 after a pre-planned interim analysis judged it unlikely to meet its primary efficacy endpoint [33], whereas OCEANIC-STROKE—testing the same asundexian 50 mg once-daily dose that proved inferior in AF—met its primary efficacy endpoint in secondary stroke prevention: among 12,327 patients randomized within 72 h of non-cardioembolic ischemic stroke or high-risk transient ischemic attack, ischemic stroke occurred in 6.2% of asundexian recipients versus 8.4% of placebo recipients (cause-specific hazard ratio 0.74; 95% CI 0.65–0.84), with no increase in ISTH major bleeding [34]. A systematic review of currently available trials [35] found an increased risk of ischemic stroke overall (odds ratio 3.37; 95% CI 2.18–5.19). Excluding OCEANIC-AF attenuated but did not abolish that signal (odds ratio 2.22; 95% CI 1.08–4.55); it was the composite of stroke or systemic embolism, significant in a fixed effect model (odds ratio 3.01; 95% CI 2.10–4.31), that became non-significant on its exclusion (odds ratio 1.52; 95% CI 0.77–3.01). Class-level conclusions therefore remain premature, and the reasons are pharmacological rather than rhetorical. The AZALEA-TIMI 71 investigators set out three grounds on which the OCEANIC-AF result should not be extrapolated to abelacimab: abelacimab binds the inactive zymogen and prevents factor XIa generation, whereas asundexian inhibits factor XIa activity only; abelacimab achieves a 99% reduction in free factor XI at the 150 mg dose, while asundexian 50 mg appears less potent; and abelacimab has proof-of-concept antithrombotic efficacy from a dedicated trial after knee arthroplasty, whereas no equivalent proof-of-concept study was performed for asundexian. The divergence between OCEANIC-AF and OCEANIC-STROKE adds a further caution: the same agent at the same dose failed in one indication and succeeded in another, so the results do not generalize across indications any more readily than across molecules. No single agent’s performance can stand for the class, and this article’s recommendations should not be read as class-level claims. For stewardship programs, the practical point is preparedness rather than adoption: should any agent report a positive phase 3 result, institutional protocols would be required for dose selection, peri-procedural management (the long half-life of a monthly monoclonal antibody is a logistical change), and bleeding management (no agent in this class has a specific reversal agent in late-phase development). We make no prediction as to whether or when any such approval will occur [Expert opinion].

### 5.6. Special Populations: Elderly, Renal, Cancer, and Obesity

Four special population contexts are considered separately below. They are commonly grouped into a single paragraph, but they differ from one another in both the strength and the kind of evidence available—from a dedicated randomized trial at one end to pharmacokinetic inference at the other—and presenting them as one block obscures exactly the distinction a stewardship protocol needs to make. Protocols should be explicit about which recommendations rest on primary trial data, which on post hoc subgroup analyses, and which on expert guidance.

#### 5.6.1. The Very Elderly

ELDERCARE-AF (Okumura et al., NEJM 2020) [29] randomized very elderly patients with AF (≥80 years) judged unsuitable for standard-dose DOAC to edoxaban 15 mg once daily or placebo, and established superiority for stroke or systemic embolism with a modest increase in bleeding. This is dedicated randomized evidence in the population to which it is applied—the strongest of the four contexts considered here—and it should prompt stewardship programs to revisit blanket “no anticoagulation” defaults in this growing group [RCT].

#### 5.6.2. Kidney Failure and Hemodialysis

The evidence here is the weakest of the four, and the uncertainty is structural rather than incidental. Two randomized trials have compared apixaban with a vitamin K antagonist in patients with AF on chronic hemodialysis, and neither settled the question. RENAL-AF [36] was stopped early after randomizing 154 of a planned 762 patients because of slow enrollment, and reported no significant difference in bleeding or thromboembolism, with confidence intervals far too wide to exclude clinically important effects in either direction. AXADIA-AFNET 8 [37] randomized patients to apixaban 2.5 mg twice daily—a dose lower than that approved for this population in the United States—or to phenprocoumon, and likewise found no difference in safety or effectiveness. In both trials, time in therapeutic range in the comparator arm was poor, so the control was arguably below standard of care, which biases the comparison in a direction that flatters apixaban. The defensible summary is therefore not that apixaban and vitamin K antagonists are equivalent in dialysis, but that the trials conducted to date are underpowered, dose-limited, and comparator-limited, and that apixaban remains a reasonable but unproven choice. Stewardship protocols should say so explicitly rather than presenting a default as though it were settled [RCT; evidence inconclusive].

#### 5.6.3. Cancer-Associated VTE

The CARAVAGGIO subgroup analysis [38] and subsequent meta-analyses reinforce apixaban as the factor Xa inhibitor of choice in cancer-associated VTE with gastrointestinal or genitourinary bleeding risk. This rests on subgroup and pooled analyses rather than on a randomized comparison designed to answer the question, and carries correspondingly less weight than the API-CAT result discussed in Section 5.2 [RCT subgroup].

#### 5.6.4. Obesity and Extremes of Body Weight

ISTH SSC guidance [39] considers any DOAC appropriate up to a body mass index of 40 kg/m^2^ or a weight of 120 kg. Above that threshold, the 2021 update suggests that standard doses of rivaroxaban or apixaban remain appropriate options, with fewer supportive data for apixaban than for rivaroxaban, while advising against dabigatran, edoxaban, and betrixaban; it also no longer recommends routine peak or trough level monitoring. The supporting evidence above the threshold is largely pharmacokinetic and observational rather than randomized, and the 40 kg/m^2^ figure should be read as a pragmatic guidance threshold rather than a biological cut-point [Guideline].

### 5.7. The Evidence Base for Stewardship Programs

The science around stewardship interventions has matured alongside the drug science, but it remains largely uncontrolled. Single-center reports describe favorable economics and improved process measures: a hemostatic and antithrombotic stewardship service returned almost six times its operating cost [40]; a prospective before-and-after evaluation in two hospitals found higher adherence to anticoagulant guidelines during the intervention period (adjusted odds ratio 1.58, 95% CI 1.21–2.05), driven mainly by low-molecular-weight heparin dosing [41]; and a full-service inpatient program incorporating a venous thromboembolism transition-of-care service reported approximately US$270,000 in savings [42]. Clinical outcomes are less well characterized. A meta-analysis of pharmacist-participated warfarin management across 24 studies found reduced total bleeding (risk ratio 0.51, 95% CI 0.28–0.94) but no significant effect on major bleeding, thromboembolism, or mortality [43], and a prospective evaluation of a multidisciplinary antithrombotic team in the same two-hospital cohort reported a progressive fall in the composite of bleeding and thrombotic events from admission to three months after discharge, amounting to a reduction of 1.83% per two-month period (95% CI 1.08% to 2.58%) [44]. Three caveats limit strong interpretation: cluster-randomized comparisons of stewardship to usual care are sparse; component attribution is rarely possible; and evidence is disproportionately from academic centers in high-income countries. The field needs cluster-randomized comparative-effectiveness trials between stewardship models rather than further uncontrolled pre–post reports.

## 6. Policy Context: Brief Implications

The scientific advances reviewed in Section 5 reach patients through health system structures that are, at present, uneven. The Joint Commission’s NPSG.03.05.01 is process-focused [45]. The CMS Quality Payment Program contains a single anticoagulation measure (Quality ID 326) [46] and retired its dedicated improvement activity on invasive procedure anticoagulation management in the 2025 final rule [47]. International exemplars (UK Quality and Outcomes Framework, Sweden’s AF registry) are primary care-focused. In Saudi Arabia, the Saudi Central Board for Accreditation of Healthcare Institutions (CBAHI) has incorporated anticoagulation-related safety standards, and the Saudi Commission for Health Specialties (SCFHS) is well positioned to embed stewardship competencies in training; DOAC availability through Ministry of Health procurement compares favorably with neighboring lower-income countries, where apixaban’s 2023 addition to the WHO Essential Medicines List [48] has not yet translated into formulary availability. Table 5 summarizes the principal policy instruments and their limitations.

## 7. Policy Adjustments to Support Translation

The five adjustments proposed in this section (Table 6) are the authors’ own proposals. They are not findings of the evidence reviewed in Section 3, Section 4 and Section 5, no trial or guideline recommends them, and they are offered as reasoned suggestions that a reader may reject while accepting everything in the preceding sections. They are labeled [Expert opinion], and Table 6 is presented on the same basis. With that stated, five modest adjustments would in our view reduce the delay between trial publication and routine uptake of the advances in Section 5. The Joint Commission NPSG.03.05.01 could include an outcome-anchored element—public reporting of DOAC dose-appropriate prescribing and major bleeding rates. CMS should restore a dedicated improvement activity for peri-procedural anticoagulation management and add measures for DOAC dose appropriateness and transition-of-care quality. Certified EHR vendors should provide validated anticoagulation decision support modules incorporating the 2023 AF risk magnitude approach. Training pathways (ACGME; SCFHS and equivalents regionally) should formalize anticoagulation stewardship competencies proportional to the clinical burden. The international thrombosis community should treat LMIC DOAC access as a genuine advocacy priority. These are implications of the clinical science rather than ends in themselves.

## 8. Open Questions the Field Must Still Answer

Several important questions remain unresolved. The factor XI class-level efficacy question will be settled by LIBREXIA-AF and LILAC-TIMI 76. The andexanet-vs-4F-PCC value question—ten-fold cost for a modest absolute hemostatic advantage and a meaningful thrombotic signal—is currently being resolved institution-by-institution, when national health technology assessment decisions would be more appropriate; UK NICE, Germany’s G-BA, and Canada’s CADTH have produced consistent restrictive guidance, the United States has not. For cancer VTE, API-CAT settles the dose question for apixaban extension but not for edoxaban or rivaroxaban, or for specific tumor biologies (primary brain tumors, genitourinary cancers) where risk profiles may differ. Global DOAC access in LMIC settings—including parts of the Middle East outside the Gulf—remains the most important unsolved problem in the field and the least often raised in clinical forums.

### Limitations of the Evidence Reviewed

Several limitations constrain what the evidence reviewed here can support, and they bear directly on how far it should travel.

Generalizability across populations. The pivotal trials underpinning this review were conducted predominantly in North America, Europe, and East Asia. COBRRA randomized patients at 32 centers in three countries; ANNEXA-I, though conducted at 131 sites in 23 countries, enrolled a population with a mean age near 79 and a heavy burden of vascular disease. Bleeding and thromboembolic risk vary with ethnicity, comorbidity, background stroke risk, and the quality of anticoagulation management, and Middle Eastern, African, and South Asian populations are underrepresented across the trial base. The effect estimates we report should be read as reliable within the populations studied and as provisional elsewhere—including in our own region, where, as Section 3 notes, contemporary surveillance of comparable size does not exist.

Generalizability across health systems. The stewardship and policy material is more system-dependent still. The instruments discussed in Section 6—Joint Commission accreditation, CMS payment mechanisms, national health technology assessment bodies—are specific to particular jurisdictions, and the evidence for stewardship interventions themselves derives disproportionately from academic centers in high-income countries with mature electronic health records and established clinical pharmacy services. Whether the same interventions produce the same effects where those preconditions are absent is untested. The reforms proposed in Section 7 are correspondingly conditional on the systems they address.

Limitations of the trial evidence itself. Much of what we report rests on single trials, and several rest on trials that were not designed to answer the question we are asking of them. COBRRA is one open-label trial excluding cancer-associated thrombosis, and its findings have not yet been incorporated into society guidance. API-CAT establishes non-inferiority within a pre-specified margin at a low absolute event rate, which is not equivalence, and settles the dose question for apixaban but not for edoxaban or rivaroxaban. ANNEXA-I used a partly management-dependent composite endpoint in an unblinded trial. AZALEA-TIMI 71 was powered for safety and cannot speak to efficacy. The hemodialysis trials are underpowered and comparator-limited. Where a conclusion rests on one such trial we have tried to say so at the point of the claim rather than only here.

Limitations of the review. This is a narrative review with the methodological constraints set out in Section 2: no registered protocol, no duplicate screening, no risk-of-bias assessment, and an interpretive selection of topics made by two authors who share a clinical background and a working context. Another pair of authors applying the same criterion might reasonably have selected differently, and the emphasis on stewardship as the mechanism of translation is itself a position rather than a finding.

## 9. Five Immediate Priorities for Stewardship Programs

Moving from framework to action, we identify five near-term priorities that stewardship programs can implement now, ordered by immediacy and likely clinical impact.

(i) Implement the 2023 AF guideline’s risk magnitude approach in institutional decision support, with particular attention to sex-specific thresholds and to validated non-CHA_2_DS_2_-VASc risk calculators for the intermediate-risk population [Guideline].

(ii) Update cancer VTE protocols to incorporate API-CAT: Prompt reassessment at the six-month mark, with reduced-dose apixaban 2.5 mg twice daily as the default for eligible patients and documentation of the basis for continuation versus de-escalation [RCT].

(iii) Standardize andexanet alfa pathways to ANNEXA-I-eligible scenarios, with 4F-PCC remaining the default for extracranial major bleeding; institute formal cost-effectiveness monitoring pending national health technology assessment guidance [RCT + institutional interpretation].

(iv) Begin protocol-readiness scenario planning for factor XI inhibitors, including dosing algorithms (monthly monoclonal vs. twice-daily oral small molecule), peri-procedural management (long half-life considerations), and education materials for anticipated phase 3 readouts in 2026–2027 [Expert opinion].

(v) Advocate, through professional societies, for the modest policy reforms outlined in Section 7—outcome-anchored accreditation, restored CMS improvement activities, formalized training competencies—with advocacy adapted to local national contexts [Expert opinion].

Key Points for Clinician:

- For AF anticoagulation: Anchor the decision on annual stroke risk magnitude, not CHA_2_DS_2_-VASc score alone. Anticoagulate at ≥2%/year (Class I); use shared decision-making at 1–2%/year; withhold anticoagulation at <1%/year. Apply sex-specific thresholds [Guideline].
- For acute non-cancer VTE: Apixaban may reasonably be preferred over rivaroxaban as the default DOAC. COBRRA [26], a superiority trial for safety, demonstrated roughly half the clinically relevant bleeding (3.3% vs. 7.1%; RR 0.46, 95% CI 0.33–0.65) and one-sixth the major bleeding (0.4% vs. 2.4%; RR 0.16, 95% CI 0.06–0.40), with no efficacy trade-off. Institutions may wish to review order sets and ED pathways accordingly [RCT; order set change = institutional interpretation].
- For extended cancer VTE prevention: Beyond six months, stepping apixaban down to 2.5 mg twice daily is an appropriate default in eligible patients—this reduces clinically relevant bleeding with no loss of efficacy (API-CAT [25], [RCT].
- For factor Xa inhibitor-associated intracerebral hemorrhage: It may be appropriate to restrict andexanet alfa to ANNEXA-I-eligible patients within the dosing window; 4F-PCC remains the default for extracranial major bleeding and for patients outside andexanet eligibility. The hemostatic advantage is real but modest (67.0% vs. 53.1%) and is offset by a meaningful thrombotic signal (10.3% vs. 5.6%) [RCT; restriction pathway = institutional interpretation].
- For factor XI inhibitors: Adoption would be premature. Class-level conclusions about efficacy remain premature; institutions may wish to consider protocol-readiness planning in anticipation of the LIBREXIA-AF and LILAC-TIMI 76 readouts [Expert opinion].
- For apixaban prescribing: Apply the three label-based reduction criteria (age ≥ 80 years, weight ≤ 60 kg, serum creatinine ≥ 1.5 mg/dL) as a set; reduce the dose only when two of three are met. Dose reduction on the basis of a single criterion is a recognizable and correctable source of thromboembolic harm [Label/Guideline; harm association from observational data].
- For the very elderly (≥80 years) with high bleeding risk: Consider edoxaban 15 mg daily as an alternative to no anticoagulation, following ELDERCARE-AF [RCT].

## 10. Closing Synthesis

Anticoagulation practice has moved substantially between 2023 and 2026. The 2023 AF guideline reframes stroke prevention around magnitude of risk. API-CAT establishes reduced-dose apixaban as the new standard for extended cancer VTE prevention. COBRRA supports apixaban as a reasonable default over rivaroxaban for acute non-cancer VTE, on the evidence of a single trial. ANNEXA-I partially resolves the factor Xa inhibitor reversal question while introducing a high-cost decision that most health systems are not structurally ready to make at a national level. The factor XI inhibitor class has delivered both its clearest failure (OCEANIC-AF) and its most encouraging safety signal (AZALEA-TIMI 71) within a twelve-month window, with pivotal phase 3 readouts ahead. For stewardship programs, the task is partly anticipatory and partly immediate: translating guideline and trial changes into consistent bedside practice. The accompanying policy reforms—outcome-anchored accreditation, restored payment program measures, standardized EHR decision support, formalized training competencies, and LMIC access mechanisms—would materially shorten the interval between trial publication and routine care. None requires controversial political action, and the case strengthens each time an important trial appears and takes years to reach community uptake. Closing the translation gap—principally through stewardship programs, supported by modest policy reform—is the practical agenda for the next three to five years.

## Figures and Tables

**Table 1:** What we see and what we miss: the categories of anticoagulant harm.

Category	Current Visibility	Policy Implication
Major bleeding	Well captured (ISTH criteria, pharmacovigilance)	Metrics adequate; benchmarking feasible
Dose-inappropriate DOAC prescribing	Detectable in EHR audits but rarely reported	Candidate for mandatory quality metric
Underprescribing (omission)	Visible in registries; not in routine reporting	Priority measure for AF and cancer VTE
Transition-of-care failures	Largely invisible—falls between inpatient and outpatient data	Requires new integrated metrics
Equity disparities	Rarely measured; never aggregated nationally	Must be embedded in all new policy from the outset
Iatrogenic thromboembolism from withholding	Invisible in bleeding-focused surveillance	Paired prescribing + outcome metrics needed

ISTH, International Society on Thrombosis and Haemostasis; VTE, venous thromboembolism.

**Table 2:** The six decision points where anticoagulation most often goes wrong.

Decision Node	Error Pattern	Stewardship Intervention
DOAC initiation in AF	Inappropriate dose reduction (apixaban 1-of-3 rule applied); rivaroxaban food instruction omitted; mechanical valve or triple-positive APS missed	CDS with forced renal/weight/age inputs; pharmacist review within 24 h
DOAC initiation for VTE	Cancer VTE decision (LMWH vs. DOAC); extended-phase dose; antiphospholipid missed	Protocolized pathway; automated duration review; APS screening trigger
Peri-procedural interruption	Unnecessary bridging; incorrect interruption interval; failed resumption	PAUSE/BRIDGE protocol integration; surgical scheduling CDS
Major bleeding event	Inappropriate reversal agent; cost-inefficient andexanet use	ED protocol; pharmacy pre-authorization for andexanet
Hospital discharge	Duplicate therapy; unclear duration; reconciliation failure	Mandatory reconciliation; standardized education; clinic handoff
Renal function change	Dose not adjusted as CrCl falls	Automated monitoring with prescriber alert

APS, antiphospholipid syndrome; CDS, clinical decision support; CrCl, creatinine clearance; ED, emergency department; LMWH, low-molecular-weight heparin; VTE, venous thromboembolism.

**Table 3:** Practice-changing updates in anticoagulation, 2023–2026.

Development	Key Finding	Change from Prior Practice	Practical Implication
2023 ACC/AHA/ACCP/HRS AF guideline (Joglar et al., JACC/Circulation 2024 [24])	Anticoagulation threshold anchored on annual risk magnitude (Class I at ≥2%/year)	Moves away from exclusive reliance on CHA_2_DS_2_-VASc cut-off; expands to ATRIA, GARFIELD-AF; sex-specific thresholds	Update EHR decision support; careful handling of the 1–2%/year intermediate zone
API-CAT (Mahé et al., NEJM 2025 [25])	Apixaban 2.5 mg BID non-inferior to 5 mg BID for extended cancer VTE (recurrence 2.1% vs. 2.8%; aSHR 0.76, 95% CI 0.41–1.41; NI margin 2.00); less bleeding (12.1% vs. 15.6%)	Establishes a step-down at the 6-month mark as a defensible default	Update cancer VTE protocols with prompted dose reassessment at 6 months
COBRRA (Castellucci et al., NEJM 2026 [26])	Apixaban vs. rivaroxaban in acute VTE: clinically relevant bleeding 3.3% vs. 7.1% (RR 0.46, 95% CI 0.33–0.65); major bleeding 0.4% vs. 2.4% (RR 0.16, 95% CI 0.06–0.40); no efficacy difference	First adequately powered randomized head-to-head comparison of two DOACs; PROBE design, superiority hypothesis for safety; non-cancer VTE only	Apixaban may reasonably be preferred as the default DOAC for acute non-cancer VTE; consider reviewing order sets and ED pathways
ANNEXA-I (Connolly et al., NEJM 2024 [27])	Andexanet hemostatic efficacy 67.0% vs. 53.1% usual care; thrombotic events 10.3% vs. 5.6%; ischemic stroke 6.5% vs. 1.5%	First randomized trial comparing andexanet to usual care (predominantly 4F-PCC)	Restrict andexanet to ANNEXA-I-eligible intracranial scenarios; 4F-PCC default otherwise
AZALEA-TIMI 71 (Ruff et al., NEJM 2025 [25])	Abelacimab 150 mg monthly vs. rivaroxaban: 62% major/CRNMB bleeding (HR 0.38, 95% CI 0.24–0.60); phase 2b safety-driven design, not powered for stroke efficacy	Strongest safety signal yet for the factor XI class	Awaits LILAC-TIMI 76 (Phase 3, OAC-unsuitable) before routine use
OCEANIC-AF (Piccini et al., NEJM 2025 [28])	Asundexian 50 mg daily vs. apixaban terminated early for inferiority (HR 3.79 for stroke/SE)	First major efficacy failure in the factor XI class	Asundexian not adopted in standard-risk AF; dose and mechanism lessons for LIBREXIA-AF
ELDERCARE-AF (Okumura et al., NEJM 2020 [29]; ongoing relevance)	Edoxaban 15 mg daily reduced stroke/SE vs. placebo in very elderly ≥80 y	Provides evidence against blanket non-anticoagulation in frail elderly	Reconsider “no anticoagulation” defaults in ≥80 y high-bleeding-risk patients

AF, atrial fibrillation; BID, twice daily; CrCl, creatinine clearance; CRNMB, clinically relevant non-major bleeding; EHR, electronic health record; HR, hazard ratio; OAC, oral anticoagulant; SE, systemic embolism; VTE, venous thromboembolism.

**Table 4:** Factor XI inhibitor program: current status.

Agent	Class/Route	Phase 3 Trial	Population	Current Status
Asundexian	Small molecule/oral once daily	OCEANIC-AF	AF vs. apixaban	Stopped early: inferior to apixaban (HR for stroke/SE 3.79)
Asundexian	Small molecule/oral	OCEANIC-AFINA	AF, unsuitable for OAC	Announced 2023; vs. placebo in OAC-ineligible population. Announced Nov 2023; recruitment never started; design under re-evaluation after OCEANIC-AF; no record of activation as of March 2026
Milvexian	Small molecule/oral twice daily	LIBREXIA-AF	AF vs. apixaban	Ongoing; >20,000 enrolled; topline data anticipated 2026
Milvexian	Small molecule/oral	LIBREXIA-ACS/-STROKE	Post-ACS, secondary stroke	LIBREXIA-ACS stopped November 2025 at interim analysis for futility; LIBREXIA-STROKE ongoing, data anticipated 2026
Abelacimab	Monoclonal antibody/monthly	LILAC-TIMI 76	AF, unsuitable for OAC	Ongoing; phase 2b AZALEA-TIMI 71 (safety-driven design, not powered for stroke efficacy) stopped early: 62% major/CRNMB bleeding vs. rivaroxaban (150 mg dose)

ACS, acute coronary syndrome; AF, atrial fibrillation; CRNMB, clinically relevant non-major bleeding; HR, hazard ratio; OAC, oral anticoagulant; SE, systemic embolism.

**Table 5:** Current policy instruments at a glance.

Instrument	Scope	Principal Limitation
Joint Commission NPSG.03.05.01	All accredited hospitals; 8 elements of performance	Process-focused; no payment consequence
CMS MIPS Quality ID 326	AF anticoagulation prescribing	Single binary measure; no quality-of-prescribing dimension
Retired CMS IA PSPA_27 (2025)	Invasive procedure/surgery management	Removed; no replacement
ASH/ACCP/ISTH/ESC guidelines	Comprehensive clinical guidance	Voluntary; variable uptake without payment alignment
UK QOF/Sweden AF-registry	Exemplary population-level tracking	Primary care-focused; hospital stewardship not addressed

AF, atrial fibrillation; CMS, Centers for Medicare & Medicaid Services; IA, improvement activity; MIPS, Merit-based Incentive Payment System; NPSG, National Patient Safety Goal; QOF, Quality and Outcomes Framework.

**Table 6:** Five targeted reforms, aligned to specific clinical opportunities.

Reform	Clinical Opportunity It Enables	Primary Stakeholder(s)
Outcome-anchored NPSG	Visible accountability for dose appropriateness, bleeding rates, transition-of-care safety	Joint Commission, JCI, CBAHI, national accreditors
Restored CMS improvement activity + new measures	Peri-procedural care, DOAC dose appropriateness, transitions (PAUSE, 2023 AF guideline, API-CAT)	CMS, commercial payers
EHR decision support standardization	Consistent implementation of 2023 AF risk magnitude approach and factor XI dosing	ONC, EHR vendors
Formalized training competencies	Trainee exposure to DOAC selection, API-CAT de-escalation, PAUSE, ELDERCARE-AF dosing, reversal algorithms	ACGME, SCFHS, specialty boards, ASHP
LMIC DOAC access program	Global translation of modern anticoagulation science, not just high-income settings	WHO, ISTH, ministries of health, philanthropies

ACGME, Accreditation Council for Graduate Medical Education; ASHP, American Society of Health-System Pharmacists; CBAHI, Saudi Central Board for Accreditation of Healthcare Institutions; CMS, Centers for Medicare and Medicaid Services; DOAC, direct oral anticoagulant; EHR, electronic health record; ISTH, International Society on Thrombosis and Haemostasis; JCI, Joint Commission International; LMIC, low- and middle-income country; NPSG, National Patient Safety Goal; ONC, Office of the National Coordinator; SCFHS, Saudi Commission for Health Specialties; WHO, World Health Organization.

## Data Availability

No new data were created or analyzed in this study. Data sharing is not applicable to this article.
